# MiRNA126 – RGS16 – CXCL12 Cascade as a Potential Mechanism of Acute Exercise-Induced Precursor Cell Mobilization

**DOI:** 10.3389/fphys.2021.780666

**Published:** 2021-12-09

**Authors:** Michelle Schmid, Helena Caria Martins, Gerhard Schratt, Julia M. Kröpfl, Christina M. Spengler

**Affiliations:** ^1^Exercise Physiology Lab, Institute of Human Movement Sciences and Sport, ETH Zurich, Zurich, Switzerland; ^2^Systems Neuroscience, Institute for Neuroscience, ETH Zurich, Zurich, Switzerland; ^3^Zurich Center for Integrative Human Physiology (ZIHP), University of Zurich, Zurich, Switzerland

**Keywords:** stem cell mobilization, CD34+/CD45dim cell mobilization, CXCL12, miRNA126, RGS16, apoptosis, oxidative stress index, acute exhaustive exercise

## Abstract

Acute exercise enhances circulating stem and precursor cells (CPCs) in the peripheral blood. The responsible mechanisms and molecular pathways, however, have not been fully identified. The aim of the present study was to investigate a pathway related to elevated levels of apoptotic peripheral blood mononuclear cells (MNCs) and their secretome. An increased uptake of miRNA126 in MNCs was suggested to lead to reduced levels of RGS16 mRNA and, in turn, an enhanced translation and secretion of CXCL12. Eighteen healthy, young men underwent two identical incremental cycling exercises of which the first served as control while the second was preceded by a 7-day-long antioxidative supplementation. Blood samples were collected at baseline (−10min) and several time points after exercise (0, 30, 90, 180, and 270min). Relative concentrations of miRNA126 in MNCs and CXCL12 levels in plasma were determined at all time points while RGS16 mRNA was assessed in MNCs at baseline and 30min after exercise. CXCL12 increased after exercise and strongly correlated with CPC numbers. MiRNA126 increased 30min and, to a lesser extent, also 180 and 270min after exercise but only with supplementation. RGS16 mRNA decreased 30min after exercise independent of the intervention. The amount of RGS16 mRNA inversely correlated with levels of miRNA126, but not with plasma CXCL12. In conclusion, even though plasma CXCL12 correlated with CPC numbers, the increase in CXCL12 cannot be explained by the increased concentration of miRNA126 and lower RGS16 mRNA in MNCs that would have allowed for an enhanced translation of CXCL12.

Clinical Trial Registration: ClinicalTrials.gov, NCT03747913. Registered 20 November 2018, https://clinicaltrials.gov/ct2/show/NCT03747913.

## Introduction

Under physiologic conditions and at rest, the human peripheral blood contains a relatively low number of circulating stem and precursor cells (CPCs, in the present publication defined as the entirety of circulating angiogenic, as well as non-angiogenic precursor cells), which are able to replenish the vessel-lining endothelium (Timmermans et al., 2009), cells of the immune, and blood system (Ratajczak, 2015), as well as mesenchymal tissues (Hass et al., 2011). Physiological stressors, such as acute exercise, have been shown to induce a transient, but substantial increase in CPC numbers in a variety of studies (Schmid et al., 2021). The detailed mechanisms as well as the extent of the interplay of different factors, however, have not yet fully been solved.

Agha et al. (2018) identified sympathetic stress as one of the responsible mechanisms for stem cell mobilization after acute exercise. Further, a study by Montgomery et al. (2019) could not confirm blood flow restriction as another potential player in exercise-dependent CPC mobilization. A recent publication of our own group studied the effects of oxidative stress evoked by a dynamic bout of exercise and examined whether a reduction in the oxidative stress index due to antecedent antioxidative supplementation could have an effect on exercise-induced CPC mobilization (Schmid et al., 2020). Our results suggested that oxidative stress was not a direct driving mechanism behind exercise-related CPC mobilization. An indirect influence of oxidative stress on CPC mobilization through the induction of apoptosis in circulating peripheral blood mononuclear cells (MNCs), however, could not be excluded. Although we did not observe any attenuation of the exercise-induced increase of apoptotic MNCs with a reduced oxidative stress level, other redundant mechanisms that induce cell apoptosis after exercise could be at play. Moreover, in our recent study, circulating precursor cell numbers significantly correlated with apoptotic MNC numbers in a repeated-measures correlation performed over all time points, which suggests that further investigations are warranted. In face of the diverse and multi-faceted roles that have already been attributed to apoptotic MNCs and their cell secretome (Beer et al., 2016), a working model of CPC mobilization after exercise that focusses on circulating apoptotic MNCs and includes players at a molecular level might be of scientific interest. The present study therefore presents a follow-up analysis to our previously published results, addressing one of these potential mechanisms (Schmid et al., 2020).

We started at the downstream end of the pathway by first identifying a possible candidate that acts as an effector, thus as a stem cell mobilizing agent in the circulation. CXC chemokine ligand 12 (CXCL12) is a potent cytokine that mediates the egress of CXC receptor 4 (CXCR4)-expressing stem and precursor cells from the bone marrow to the periphery (Sugiyama et al., 2006; Yin et al., 2007). CXCL12 serum and plasma concentrations reportedly increased after various dynamic exercise interventions (Zaldivar et al., 2007; Van Craenenbroeck et al., 2010, 2011; Chang et al., 2015; Niemiro et al., 2017, 2018).

MicroRNAs (miRNAs) are small, non-coding RNAs that function as highly conserved regulators of gene expression by recognizing specific sequences on their messenger RNA (mRNA) targets, inducing mRNA degradation or repression of protein translation (Cai et al., 2009). MiRNA126 is primarily expressed by endothelial cells and has previously been shown to be present in plasma in increased concentrations after physical exertion (Uhlemann et al., 2014). A study by Zernecke et al. (2009) found miRNA126 to be abundantly present in apoptotic bodies derived from human umbilical vein endothelial cells *in vitro*. Additionally, miRNA126-containing bodies were shown to be taken up by recipient cells where they upregulated CXCL12 expression *via* control of the regulator of G protein signaling 16 (RGS16), a potent suppressor of CXCL12 (Zernecke et al., 2009). We speculated that these findings might also translate to MNCs in a human *in vivo* setting where increased apoptotic MNC counts after an acute bout of exercise could also release increased amounts of miRNA126-containing bodies. In turn, they might be taken up by intact MNCs, inducing a heightened production, and release of CXCL12 into circulation, as freshly isolated lymphocytes and monocytes have previously been shown to secrete CXCL12 *in vitro* (Sánchez-Martín et al., 2011).

Thus, the aim of the present investigation was (1) to characterize CPC mobilization on a molecular level by looking at a potential pathway connected to the apoptotic MNC secretome and (2) to examine whether the extent of exercise-induced oxidative stress plays a significant role for this pathway as an apoptosis-inducing element.

We hypothesized that increased numbers of CPCs after acute exercise are induced by an elevated concentration of circulating CXCL12, which would be caused by lower levels of RGS16 mRNA due to miRNA126 uptake in MNCs. The kinetics of this cascade should correlate with the extent of cell apoptosis and be connected to oxidative stress.

## Materials and Methods

### Ethics Approval

This study was approved by the Ethics Committee of the Canton of Zurich (project ID: BASEC 2018-02075) and registered at clinicaltrials.gov (NCT03747913), as well as at the Swiss National Clinical Trials Portal (SNCTP). Written informed consent was obtained from all participants.

### Participants and Study Design

This investigation is part of a larger project (Schmid et al., 2020) and presents an exclusive follow-up analysis to further explain and investigate previous findings in more detail. Results of the present investigation address completely novel aspects and have not yet been addressed before. Participant characteristics and details of the study design have been described in more detail previously (Schmid et al., 2020).

In brief, 18 healthy, young, and normal-weight male participants (18–35years) performed two identical, individually standardized incremental cycling sessions, starting at 20% of their individual maximal workload, increasing by 20% every 2min up to 100% of their individual maximal workload determined in a prior maximal incremental cycling test to exhaustion. Prior to the second exercise session, participants underwent a 1-week-long supplementation of antioxidative vitamins (Vitamin C retard; 2×500mg/day; Burgerstein Vitamine, Antistress AG, Rapperswil-Jona, Switzerland and Vitamin E; 400 I.E/day, Burgerstein Vitamine).

### Blood Withdrawal and Processing

Venous blood was drawn by repeated venepuncture 10min before (baseline) and at several time points after exercise cessation (0, 30, 90, 180, and 270min) from the antecubital vein into 6ml BD vacutainer tubes spray-coated with EDTA anti-coagulant for isolation of MNCs and 2ml BD vacutainer tubes spray-coated with EDTA anti-coagulant for isolation of plasma (Becton Dickinson AG, Allschwil, Switzerland).

Plasma was isolated by immediate centrifugation (1,500g, 10min at room temperature) and stored in aliquots at −80°C until further analysis of CXCL12 concentrations. MNCs were isolated using Ficoll Histopaque-dependent density gradient centrifugation (Ficoll-Paque™_Plus; GE Healthcare, Opfikon, Switzerland). Isolated MNCs were lysed in 1ml peqGOLD TriFast™ (VWR, Dietikon, Switzerland) and stored at −20°C until RNA isolation and subsequent real time PCR (qPCR) analyses.

### CXC Chemokine Ligand 12 ELISA

To measure CXCL12 levels in plasma, a commercial human CXCL12/SDF-1𝛼 ELISA Kit was used (Quantikine^®^ kit DSA00, R&D systems, Bio-Techne AG, Zug, Switzerland). Analysis was performed in duplicates with 50μl of plasma and the protocol was designed according to the manufacturer’s instruction. Plasma samples were not subjected to a dual-centrifugation process in order to prevent a potential loss of platelet-bound CXCL12.

Changes in plasma volume with exercise were calculated using the calculation of Dill and Costill (1974).

### RNA Isolation and DNase Treatment

Samples of already homogenized MNCs were subjected to phase separation, RNA precipitation, washing, and solubilization. TURBO DNase treatment (Life Technologies Europe BV, Zug, Switzerland) was performed using 1μg of isolated RNA and samples were frozen at −80°C until further analysis. All steps were performed according to the manufacturers’ protocols.

### Real Time PCR Analysis

#### Relative Quantification of miRNA126 in Circulating MNCs

TaqMan™ Advanced miRNA cDNA Synthesis Kit (Thermo Fisher Scientific, Basel, Switzerland) was used to transcribe mature miRNA present in the RNA samples isolated from MNCs into cDNA. All steps were performed according to the manufacturer’s protocol. Briefly, 10μg RNA was subjected to elongation of mature miRNAs at the 3′-end *via* poly-A-tailing and ligation of a universal adaptor to the 5′-end. Samples were then reverse transcribed using universal RT primers. To enhance detection of low-expressing miRNA targets, the transcribed cDNA was further amplified.

Real time PCR was performed in triplicates for each measured miRNA and time point, using TaqMan™ Fast Advanced Master Mix (Thermo Fisher Scientific, Basel, Switzerland) and commercial primers for human miRNA126-3p and human miRNA191-5p as an endogenous control (TaqMan™ Advanced miRNA Assay; Thermo Fisher Scientific, Basel, Switzerland). Obtained data were analyzed using the Bio-Rad CFX Maestro 1.1 Software vs.4.1.2433.1219 (^©^ Bio-Rad Laboratories). Individual measurements were excluded if they deviated ≥1cycle from the other replicates or showed cycle numbers ≥35. Relative quantification of miRNA126 to miRNA191 was computed with the ΔCt method.

#### Relative Quantification of RGS16 mRNA in Circulating MNCs

MNCs isolated from samples taken at baseline and 30min after exercise cessation were analyzed for their relative expression of RGS16 mRNA. Synthesis of cDNA was performed on 200ng of DNase-treated RNA isolated from MNCs using the iScript™ cDNA Synthesis Kit (Bio-Rad Laboratories, Cressier, Switzerland) according to the manufacturer’s instructions.

iTaq™ Universal SYBR^®^ Green Supermix (Bio-Rad Laboratories, Cressier, Switzerland) and custom-made primers for RGS16 mRNA and GAPDH mRNA as an endogenous control (Thermo Fisher Scientific, Basel, Switzerland; Table 1) were used for qPCR analysis. Samples were tested in triplicates and results were analyzed as described before, using the ΔCT method. One sample had to be excluded from further analysis due to technical difficulties during preparation.

**Table 1:** mRNA primer sequences for qPCR analysis.

mRNA Target	Sequence Forward Primer	Sequence Reverse Primer
RGS16	5′-TCA CAC ACC TGA GTC TCC ACG-3′	5′-CAA CCT CTC TTC CCG CTG G-3′
GAPDH	5′-GTC TCC TCT GAC TTC AAC AGC G-3′	5′-ACC ACC CTG TTG CTG TAG CCA A-3′

### Oxidative Stress Index, Apoptotic MNC, and CPC Counts

Methods regarding the measurements of the oxidative stress index *via* total (anti-)oxidative capacities as well as the quantification of (apoptotic) MNCs and CPCs (angiogenic and non-angiogenic precursor cells) *via* flow cytometry were published previously (Schmid et al., 2020).

### Statistics

Data of CXCL12 plasma concentrations and relative quantification of miRNA126 in MNCs were analyzed using a two-way repeated-measures analysis of variance. Upon detection of significant effects, *post-hoc* tests were carried out in the form of multiple comparisons, applying Dunnett’s correction for multiple testing. Results from the relative quantification of RGS16 mRNA in MNCs were analyzed by fitting a mixed-effects model, rather than performing a repeated-measures ANOVA, due to a missing value. Multiple comparisons with Sidak’s correction were carried out in a *post-hoc* testing upon detection of significance. Mauchly’s test of sphericity was conducted on all data sets and whenever assumption of sphericity was violated, the Greenhouse–Geisser correction was applied.

To test for associations between datasets, repeated-measures correlations were carried out. The design of this test, which includes all measured time points, respects the assumption of independence and assures greater statistical power compared to simple correlations.

To test whether changes in plasma volume after exercise were significant, two-tailed one-sample t-tests were performed and the Bonferroni method was applied to correct for multiple testing.

The level of significance was set at *p*<0.05 and data are presented as mean±SD, unless indicated otherwise. All statistical tests were conducted in GraphPad Prism 9 for macOS vs. 9.0.0 (86; GraphPad Software Inc., San Diego, California Unites States), except for Mauchly’s test of sphericity which was carried out in IBM SPSS Software version 26 (IBM Corp., Armonk, NY, United States) and repeated-measures correlations which were computed in R version 4.0.5 (R Core Team, 2019; R: A language and environment for statistical computing. R Foundation for Statistical Computing, Vienna, Austria).

## Results

### Plasma Concentrations of CXCL12

**Figure 1:**
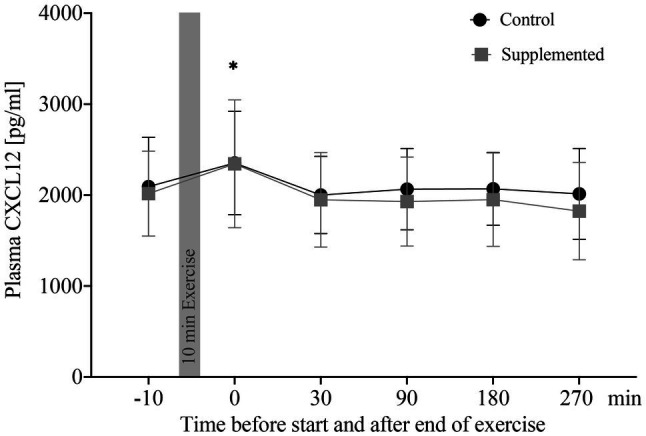
Plasma CXCL12 concentrations in pg/ml (*n*=18). Values are mean±SD. Differences from baseline are indicated by ^*^, with ^*^p<0.05.

Concentrations of plasma CXCL12 are depicted in Figure 1. The two-way ANOVA showed a significant main effect of time [*F*(3.36, 57.09)=8.532, *p*<0.0001], while intervention and interaction effects were not significant [*F*(1, 17)=2.301, *p*=0.148 and *F*(3.20, 54.35)=0.978, *p*=0.414]. *Post-hoc* analysis revealed a significant increase in CXCL12 levels from baseline to 0min after exercise in the control trial, as well as in the supplementation trial (*p*=0.040 and *p*=0.031). Overall changes in plasma volume between baseline and 0min after exercise were not significant (Δ=−0.00548, *p*=0.193, data not shown).

Correlations of plasma CXCL12 concentrations were performed for both exercise sessions with CPC numbers (entirety of angiogenic, as well as non-angiogenic precursor cells), oxidative stress index, and apoptotic MNCs. These data were published previously by our group (Schmid et al., 2020). Analysis of CXCL12 concentration and absolute CPC numbers resulted in a significant correlation for both, the control and supplementation trial (*r*=0.404, *p*<0.0001 and *r*=0.440, *p*<0.0001). Similarly, CXCL12 concentrations were significantly correlated with numbers of apoptotic MNCs in both control and supplementation trial (*r*=0.390, *p*<0.001 and *r*=0.340, *p*=0.001). The correlation between CXCL12 concentrations and oxidative stress index, however, was only significant in the control trial (*r*=0.275, *p*=0.008), while no significance was detected in the supplementation trial (*r*=0.139, *p*=0.190).

To summarize, plasma CXCL12 levels were elevated directly after exercise cessation in both interventions and CXCL12 significantly correlated with numbers of apoptotic MNCs and CPCs in circulation over all time points.

### Relative Quantification of miRNA126 in MNCs

**Figure 2:**
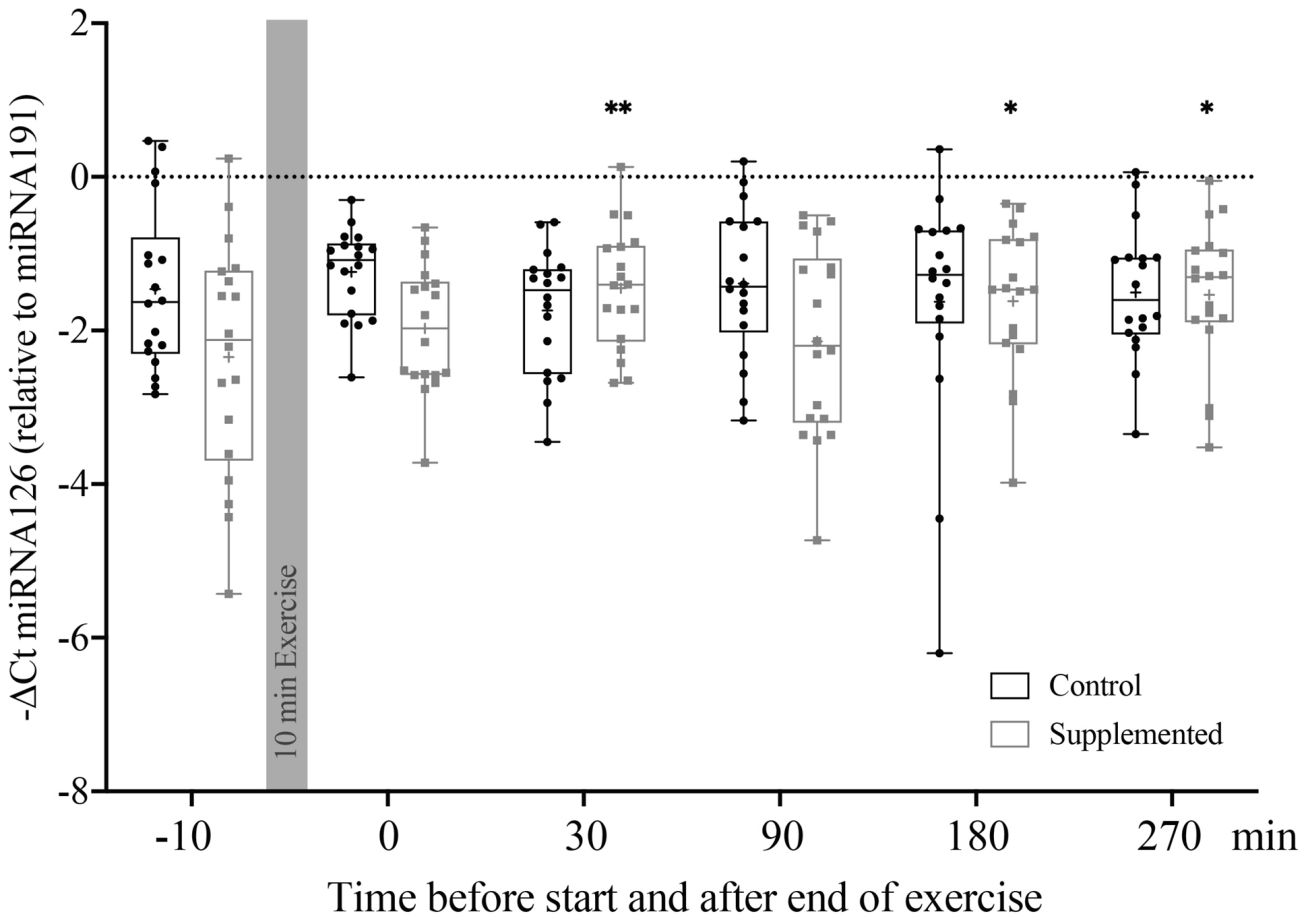
Relative quantification of miRNA126 to miRNA191 in MNCs using the ΔCt method (*n*=18). Bars include 50% of all data points while the line within the bars represents the median. Additionally, the mean is indicated by a “+”-sign in each bar. Whiskers represent minimum and maximum values with all individual data points being shown. Differences from baseline are indicated by ^*^, with ^*^p<0.05; ^**^p<0.01.

Relative Quantification of miRNA126, represented by the −ΔCt value, is shown in Figure 2. Statistical analysis *via* two-way ANOVA revealed a significant effect of interaction [*F*(5, 170)=3.506, *p*=0.005], but no significant time or intervention effects [*F*(5, 170)=1.120, *p*=0.352 and *F*(1, 34)=1.938, *p*=0.173]. Multiple comparisons with Dunnett’s correction showed significant increases in miRNA126 levels compared to baseline at 30min (*p*=0.004), 180min (*p*=0.029), and 270min (*p*=0.012) after exercise in the supplementation trial only.

To summarize, the first and most distinct increase in miRNA126 levels in MNCs was observed 30min after exercise cessation, but only in the supplementation trial.

### Relative Quantification of RGS16 mRNA in MNCs

**Figure 3:**
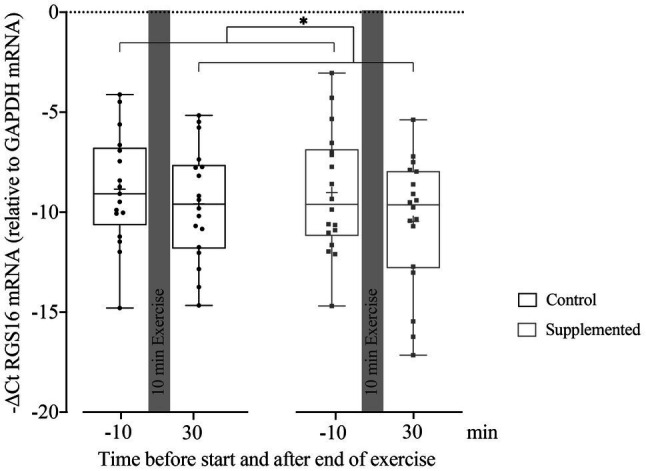
Relative quantification of RGS16 mRNA to GAPDH mRNA in MNCs using the ΔCt method (*n*=17 for control intervention and *n*=18 for supplemented intervention). Bars include 50% of all data points while the line within the bars represents the median. Additionally, the mean is indicated by a “+”-sign in each bar. Whiskers represent minimum and maximum values with all individual data points being shown. Differences between the two time points are indicated by ^*^, with ^*^p<0.05.

Quantification of RGS16 mRNA in MNCs at baseline and 30min after exercise (when the first and most substantial increase in miRNA126 levels was observed) is given in Figure 3. A mixed-effect analysis showed a significant effect of time [*F*(1, 33)=5.410, *p*=0.026] but no significance of intervention or interaction effects [*F*(1, 34)=0.405, *p*=0.529 and *F*(1, 33)=0.448, *p*=0.508]. However, the follow-up analysis of RGS16 mRNA levels 30min after exercise compared to baseline resulted in no significant difference in the control trial (*p*=0.445) but showed a trend toward reduced levels in the supplementation trial (*p*=0.078).

RGS16 mRNA levels negatively and significantly correlated with miRNA126 concentrations in the control (*r*=−0.677, *p*=0.002), as well as in the supplementation trial (*r*=−0.569, *p*=0.011). However, no significant correlation was found between RGS16 mRNA and plasma CXCL12 levels in both interventions (control trial: *r*=0.421, *p*=0.082 and supplemented trial: *r*=−0.156, *p*=0.524).

To summarize, pooled RGS16 mRNA levels in MNCs significantly decreased from pre- to 30min post-exercise. If analyzed individually, RGS16 showed a trend toward reduced levels 30min after exercise only in the supplementation trial. Over all time points, RGS16 inversely correlated with miRNA126 but not CXCL12.

## Discussion

The current investigation tested the presence of a potential pathway of stem cell mobilization after physical exertion that depends on apoptotic MNCs and their secretome involving miRNA126, RGS16, and the chemoattractant CXCL12 as main agonists.

We found that plasma CXCL12 increased after exercise and strongly correlated with CPC and apoptotic MNC numbers. The possibility of an artificial elevation of CXCL12 due to a reduced plasma volume immediately after exercise was excluded. MiRNA126 concentrations increased in MNCs 30min and, to a lesser extent, also 180 and 270min after exercise, but all three increases were only apparent in the presence of antioxidative supplementation. RGS16 mRNA levels in MNCs decreased 30min post-exercise with no detectable intervention or interaction effect. RGS16 inversely correlated with miRNA126, but not with plasma CXCL12.

Our observations of increased circulating CXCL12 directly after exercise are in line with previous publications (Zaldivar et al., 2007; Van Craenenbroeck et al., 2010, 2011; Chang et al., 2015; Niemiro et al., 2017, 2018) while no significant changes in plasma CXCL12 were found by others (Ribeiro et al., 2017; Ross et al., 2017). We assume those discrepancies might most likely result from the different nature of the physical interventions. Resistance exercise (Ribeiro et al., 2017) might not affect CXCL12 levels in the same way as an aerobic exercise intervention which lead to a significant increase directly after exercise cessation and similarly, a submaximal exercise (Ross et al., 2017) might not have been of an intensity high enough to elicit comparable increases in CXCL12 as seen after a maximal exercise intervention. Some authors additionally correlated the observed increase in CXCL12 concentrations with changes in CPCs where most did not find a significant correlation (Van Craenenbroeck et al., 2011; Niemiro et al., 2017, 2018) except for one study in young men (Ross et al., 2017). While these studies performed simple correlations on calculated changes in CPC numbers pre- to post-exercise, we detected a significant correlation between CXCL12 and CPCs by correlating absolute numbers for all measured time points in a repeated-measures design. Further, CXCL12-measurements in the present study were performed in plasma that still contained platelets while the other studies used platelet-free plasma or serum. The present study thus expands the existing knowledge in showing that CPC numbers are likely connected to (platelet-bound) plasma CXCL12 in a more general, not necessarily exercise-dependent way. A similar association was already shown for patients with congestive heart failure (Jorbenadze et al., 2014) and may also hold true in healthy participants. Furthermore, our results indicate that differences in baseline values, which are not taken into account when correlating changes, might be of importance as well. The exercise-induced increase in circulating CXCL12 observed in this and other studies might thus contribute to, but not be the only factor causing the simultaneously observed increase in CPCs.

Moreover, we showed that CXCL12 significantly correlated with apoptotic MNC numbers, regardless of the level of oxidative stress. The oxidative stress index, however, correlated with CXCL12 concentrations only if no exogenous antioxidants were supplied. This finding is consistent with our previously published results of no direct correlation between oxidative stress and the extent of apoptosis after exercise (Schmid et al., 2020), which points toward an involvement of additional, possibly redundant apoptosis-inducing mechanisms like a rise in intra-cellular Ca^2+^, growth-factor withdrawal, increases in glucocorticoid secretion, and/or inflammatory processes in general. These processes were previously seen to be associated with the induction of apoptosis in lymphocytes during and after acute exercise (Phaneuf and Leeuwenburgh, 2001; Krüger and Mooren, 2014).

In a second step, we aimed to explain the connection between levels of CXCL12 and apoptotic MNCs in circulation in analogy to a previous finding in endothelial cells where endothelial-cell derived apoptotic bodies, abundantly containing miRNA126, were taken up by vessel-lining, intact recipient endothelial cells causing them to produce increased levels of CXCL12 (Zernecke et al., 2009). We speculated that the same cause of events might also take place in MNCs where intact circulating cells take up miRNA126-containing apoptotic bodies formed by apoptotic MNCs causing them to produce and secrete enhanced amounts of CXCL12. We found an increase in miRNA126 in MNCs 30min after exercise, which is concomitant with an increase in apoptotic cells. This suggests that not only cells of the endothelium, but also MNCs hold increased levels of miRNA126 when higher numbers of apoptotic cells are present. It might be speculated that this rise is caused by an enhanced endogenous production of miRNA126 rather than the uptake of external miRNA126. We tend to reject this theory though as miRNA126 is specifically expressed by endothelial cells which make up only a minority of circulating MNCs (Fish et al., 2008; Fish and Srivastava, 2009; Schmid et al., 2020). Additionally, an enhanced uptake of miRNA126 by MNCs due to higher circulating miRNA126 levels is in line with a previous finding of significantly higher miRNA126 serum levels after exercise (Uhlemann et al., 2014). Interestingly, we only observed increased miRNA126 levels in MNCs in the supplementation trial while a previous study found increased levels of serum miRNA126 30min after exercise only without previous antioxidative supplementation (Da Silva et al., 2015). Possibly, oxidative stress impedes the cellular uptake of circulating miRNA126, which could explain increased miRNA126 in MNCs with antioxidative supplementation and increased serum miRNA126 after exercise without antioxidative supplementation. It needs to be pointed out, however, that the study by Da Silva et al. (2015) was performed in patients with intermittent claudication and findings might thus not be comparable to the situation in healthy participants.

In a last step, we examined whether the increase in miRNA126 30min after exercise translated into reduced amounts of RGS16 mRNA, a direct target of miRNA126, and, *via* its actions on CXCR4, a potent repressor of CXCL12 (Zernecke et al., 2009). According to our hypothesis, RGS16 mRNA in MNCs would be decreased 30min after exercise in the supplementation trial, which was indeed the case 30min after exercise compared to baseline, but with no significant intervention or interaction effect. Individual *post-hoc* tests revealed no significant change in RGS16 in the control trial but showed a trend toward reduced levels after exercise in the supplementation trial. Together with the correlation between RGS16 and miRNA126 levels, our data are thus consistent with the theory of RGS16 as a direct target of miRNA126 in MNCs. However, contrary to our hypothesis, lower levels of RGS16 mRNA did not result in higher levels of circulating CXCL12, neither 30min post-exercise nor at any other time point. The only significant increase in CXCL12 occurred before any changes in the miRNA126-RGS16 cascade were observed. It is thus not surprising that RGS16 mRNA and plasma CXCL12 were unrelated. Even if lower amounts of RGS16 resulted in an increased secretion of CXCL12 by MNCs, we suspect that this contribution to the general pool of CXCL12 might simply be too small to result in a physiologically significant change. This is in line with findings reported in our previous publication where we observed increased absolute numbers of apoptotic MNCs after acute exercise, but relative to the increase in total MNCs the apoptotic subset actually became smaller (Schmid et al., 2020). Our findings further agree with interpretations from a large dataset where 29 cell types within the MNC fraction were characterized by RNA sequencing, showing that RGS16 mRNA is expressed rather abundantly, while CXCL12 mRNA is expressed at very low levels and only in a few subtypes of MNCs (Monaco et al., 2019). They also indicate that the observed increase in CXCL12 levels directly after exercise is likely caused by an enhanced production and/or secretion into the peripheral blood by other tissues, such as the thymus, the lymph nodes, or the liver, which were previously shown to express CXCL12 (Sánchez-Martín et al., 2011). Future studies should investigate whether the general regulation of CXCL12 secretion of those tissues to the peripheral blood is mediated by the apoptotic MNC secretome.

We conclude that the correlation between CXCL12 and apoptotic MNCs cannot be explained by an increased uptake of miRNA126 by circulating MNCs and lower intra-cellular amounts of RGS16, allowing for an enhanced translation of CXCL12. However, we found a significant correlation of CXCL12 with CPC numbers over all measured time points. This highlights CXCL12 as an attractive target to discern other regulatory pathways of CPC numbers in the peripheral blood, also, but not only, after exercise. Additionally, we revealed an interesting connection between exercise, oxidative stress, and the miRNA126-RGS16 cascade in circulating MNCs. Even though this pathway did not translate into higher levels of circulating CXCL12 as expected, we suggest it warrants further research, possibly also examining other downstream effectors and targets of RGS16 and/or miRNA126.

### Limitations

Being a follow-up investigation of samples collected in a previous study to allow further mechanistic insight, this publication comes with certain limitations. First, we could only further investigate variables in already collected plasma samples and total MNCs. As we did not measure any apoptotic bodies or vesicles in the blood stream, our predications on their presence or uptake are limited. Also, CXCL12 mRNA concentrations in MNCs were not analyzed, which would have provided a valuable link between RGS16 mRNA and plasma CXCL12 levels. We undertook different analytical approaches but were not able to retrieve this data – most probably due to the aforementioned low levels of CXCL12 mRNA in MNCs, which fall below qPCR detection levels.

## Data Availability Statement

The original contributions presented in the study are included in the article/supplementary material, and further inquiries can be directed to the corresponding author.

## Ethics Statement

The studies involving human participants were reviewed and approved by the Ethics Committee of the Canton of Zurich. The patients/participants provided their written informed consent to participate in this study.

## Author Contributions

CS, JK, MS, HM, and GS designed the study. MS collected the data. MS, HM, and JK analyzed the data. MS, JK, HM, and CS discussed the data and wrote the manuscript. GS revised the manuscript. All authors read and approved the final manuscript.

## Funding

This research was funded by the ETH Zurich (grant ETH-43 15-2 to CS and JK).

## Conflict of Interest

The authors declare that the research was conducted in the absence of any commercial or financial relationships that could be construed as a potential conflict of interest.

## Publisher’s Note

All claims expressed in this article are solely those of the authors and do not necessarily represent those of their affiliated organizations, or those of the publisher, the editors and the reviewers. Any product that may be evaluated in this article, or claim that may be made by its manufacturer, is not guaranteed or endorsed by the publisher.

## References

[ref1] Agha N. H. Baker F. L. Kunz H. E. Graff R. Azadan R. Dolan C.. (2018). Vigorous exercise mobilizes CD34+ hematopoietic stem cells to peripheral blood via the β2-adrenergic receptor. Brain Behav. Immun. 68, 66–75. doi: 10.1016/j.bbi.2017.10.001, PMID: 29017969 PMC6980177

[ref2] Beer L. Mildner M. Gyöngyösi M. Ankersmit H. J. (2016). Peripheral blood mononuclear cell secretome for tissue repair. Apoptosis 21, 1336–1353. doi: 10.1007/s10495-016-1292-8, PMID: 27696124 PMC5082595

[ref3] Cai Y. Yu X. Hu S. Yu J. (2009). A brief review on the mechanisms of miRNA regulation. Genomics Proteomics Bioinformatics 7, 147–154. doi: 10.1016/S1672-0229(08)60044-3, PMID: 20172487 PMC5054406

[ref4] Chang E. Paterno J. Duscher D. Maan Z. N. Chen J. S. Januszyk M.. (2015). Exercise induces SDF-1 mediated release of endothelial progenitor cells with increased Vasculogenic function. Plast. Reconstr. Surg. 135:340. doi: 10.1097/PRS.0000000000000917 PMC4311572 25626819

[ref5] Da Silva N. D. Jr. Roseguini B. T. Chehuen M. Fernandes T. Mota G. F. Martin P. K.. (2015). Effects of oral N-acetylcysteine on walking capacity, leg reactive hyperemia, and inflammatory and angiogenic mediators in patients with intermittent claudication. Am. J. Phys. Heart Circ. Phys. 309, H897–H905. doi: 10.1152/ajpheart.00158.2015 26116711

[ref6] Dill D. B. Costill D. L. (1974). Calculation of percentage changes in volumes of blood, plasma, and red cells in dehydration. J. Appl. Physiol. 37, 247–248. doi: 10.1152/jappl.1974.37.2.247, PMID: 4850854

[ref7] Fish J. E. Santoro M. M. Morton S. U. Yu S. Yeh R.-F. Wythe J. D.. (2008). miR-126 regulates angiogenic signaling and vascular integrity. Dev. Cell 15, 272–284. doi: 10.1016/j.devcel.2008.07.008, PMID: 18694566 PMC2604134

[ref8] Fish J. E. Srivastava D. (2009). MicroRNAs: opening a new vein in angiogenesis research. Sci. Signal. 2:pe1. doi: 10.1126/scisignal.252pe1 19126861 PMC2680274

[ref9] Hass R. Kasper C. Böhm S. Jacobs R. (2011). Different populations and sources of human mesenchymal stem cells (MSC): a comparison of adult and neonatal tissue-derived MSC. Cell Commun. Signal. 9, 1–14. doi: 10.1186/1478-811X-9-12 21569606 PMC3117820

[ref10] Jorbenadze R. Schleicher E. Bigalke B. Stellos K. Gawaz M. (2014). Expression of platelet-bound stromal-cell derived factor-1 (SDF-1) and number of CD34+ progenitor cells in patients with congestive heart failure. Platelets 25, 409–415. doi: 10.3109/09537104.2013.829913, PMID: 24102302

[ref11] Krüger K. Mooren F. C. (2014). Exercise-induced leukocyte apoptosis. Exerc. Immunol. Rev. 20, 117–134. PMID: 24974724

[ref12] Monaco G. Lee B. Xu W. Mustafah S. Hwang Y. Y. Carre C.. (2019). RNA-Seq signatures normalized by mRNA abundance allow absolute deconvolution of human immune cell types. Cell Rep. 26, 1627.e7–1640.e7. doi: 10.1016/j.celrep.2019.01.041, PMID: 30726743 PMC6367568

[ref13] Montgomery R. Paterson A. Williamson C. Florida-James G. Ross M. D. (2019). Blood flow restriction exercise attenuates the exercise-induced endothelial progenitor cell response in healthy, young men. Front. Physiol. 10:447. doi: 10.3389/fphys.2019.00447, PMID: 31057427 PMC6478759

[ref14] Niemiro G. M. Edwards T. Barfield J. Beals J. W. Broad E. M. Motl R. W.. (2018). Circulating progenitor cell response to exercise in wheelchair racing athletes. Med. Sci. Sports Exerc. 50, 88–97. doi: 10.1249/MSS.0000000000001402, PMID: 28806276

[ref15] Niemiro G. M. Parel J. Beals J. Van Vliet S. Paluska S. A. Moore D. R.. (2017). Kinetics of circulating progenitor cell mobilization during submaximal exercise. J. Appl. Physiol. 122, 675–682. doi: 10.1152/japplphysiol.00936.2016, PMID: 28082336

[ref16] Phaneuf S. Leeuwenburgh C. (2001). Apoptosis and exercise. Med. Sci. Sports Exerc. 33, 393–396. doi: 10.1097/00005768-200103000-00010 11252065

[ref17] Ratajczak M. (2015). A novel view of the adult bone marrow stem cell hierarchy and stem cell trafficking. Leukemia 29, 776–782. doi: 10.1038/leu.2014.346, PMID: 25486871 PMC4396402

[ref18] Ribeiro F. Ribeiro I. P. Gonçalves A. C. Alves A. J. Melo E. Fernandes R.. (2017). Effects of resistance exercise on endothelial progenitor cell mobilization in women. Sci. Rep. 7, 1–9. doi: 10.1038/s41598-017-18156-6 29259281 PMC5736626

[ref19] Ross M. D. Malone E. M. Simpson R. Cranston I. Ingram L. Wright G. P.. (2017). Lower resting and exercise-induced circulating angiogenic progenitors and angiogenic T cells in older men. Am. J. Phys. Heart Circ. Phys. 314, 392–402. doi: 10.1152/ajpheart.00592.2017 29167123

[ref20] Sánchez-Martín L. Estecha A. Samaniego R. Sánchez-Ramón S. Vega M. Á. Sánchez-Mateos P. (2011). The chemokine CXCL12 regulates monocyte-macrophage differentiation and RUNX3 expression. Blood 117, 88–97. doi: 10.1182/blood-2009-12-258186 20930067

[ref21] Schmid M. Gruber H.-J. Kröpfl J. M. Spengler C. M. (2020). Acute exercise-induced oxidative stress does not affect immediate or delayed precursor cell mobilization in healthy young males. Front. Physiol. 11:1314. doi: 10.3389/fphys.2020.577540 PMC7606978 33192581

[ref22] Schmid M. Kröpfl J. Spengler C. (2021). Changes in circulating stem and progenitor cell numbers following acute exercise in healthy human subjects: a systematic review and meta-analysis. Stem Cell Rev. Rep. 17, 1091–1120. doi: 10.1007/s12015-020-10105-7, PMID: 33389632 PMC8316227

[ref23] Sugiyama T. Kohara H. Noda M. Nagasawa T. (2006). Maintenance of the hematopoietic stem cell pool by CXCL12-CXCR4 chemokine signaling in bone marrow stromal cell niches. Immunity 25, 977–988. doi: 10.1016/j.immuni.2006.10.016, PMID: 17174120

[ref24] Timmermans F. Plum J. Yöder M. C. Ingram D. A. Vandekerckhove B. Case J. (2009). Endothelial progenitor cells: identity defined? J. Cell. Mol. Med. 13, 87–102. doi: 10.1111/j.1582-4934.2008.00598.x, PMID: 19067770 PMC3823038

[ref25] Uhlemann M. Möbius-Winkler S. Fikenzer S. Adam J. Redlich M. Möhlenkamp S.. (2014). Circulating microRNA-126 increases after different forms of endurance exercise in healthy adults. Eur. J. Prev. Cardiol. 21, 484–491. doi: 10.1177/2047487312467902, PMID: 23150891

[ref26] Van Craenenbroeck E. M. Beckers P. J. Possemiers N. M. Wuyts K. Frederix G. Hoymans V. Y.. (2010). Exercise acutely reverses dysfunction of circulating angiogenic cells in chronic heart failure. Eur. Heart J. 31, 1924–1934. doi: 10.1093/eurheartj/ehq058, PMID: 20299351

[ref27] Van Craenenbroeck E. M. Bruyndonckx L. Van Berckelaer C. Hoymans V. Y. Vrints C. J. Conraads V. M. (2011). The effect of acute exercise on endothelial progenitor cells is attenuated in chronic heart failure. Eur. J. Appl. Physiol. 111, 2375–2379. doi: 10.1007/s00421-011-1843-1, PMID: 21290145

[ref28] Yin Y. Huang L. Zhao X. Fang Y. Yu S. Zhao J.. (2007). AMD3100 mobilizes endothelial progenitor cells in mice, but inhibits its biological functions by blocking an autocrine/paracrine regulatory loop of stromal cell derived factor-1 *in vitro*. J. Cardiovasc. Pharmacol. 50, 61–67. doi: 10.1097/FJC.0b013e3180587e4d, PMID: 17666917

[ref29] Zaldivar F. Eliakim A. Radom-Aizik S. Leu S.-Y. Cooper D. M. (2007). The effect of brief exercise on circulating CD34+ stem cells in early and late pubertal boys. Pediatr. Res. 61, 491–495. doi: 10.1203/pdr.0b013e3180332d36, PMID: 17515877

[ref30] Zernecke A. Bidzhekov K. Noels H. Shagdarsuren E. Gan L. Denecke B.. (2009). Delivery of microRNA-126 by apoptotic bodies induces CXCL12-dependent vascular protection. Sci. Signal. 2:81. doi: 10.1126/scisignal.2000610 19996457

